# Do Active Surveillance and Contact Precautions Reduce MRSA Acquisition? A Prospective Interrupted Time Series

**DOI:** 10.1371/journal.pone.0058112

**Published:** 2013-03-21

**Authors:** Caroline Marshall, Michael Richards, Emma McBryde

**Affiliations:** Victorian Infectious Diseases Service, Royal Melbourne Hospital and Department of Medicine, University of Melbourne, Parkville, Victoria, Australia; University of Calgary, Canada

## Abstract

**Background:**

Consensus for methicillin-resistant *Staphylococcus aureus* (MRSA) control has still not been reached. We hypothesised that use of rapid MRSA detection followed by contact precautions and single room isolation would reduce MRSA acquisition.

**Methods:**

This study was a pre-planned prospective interrupted time series comparing rapid PCR detection and use of long sleeved gowns and gloves (contact precautions) plus single room isolation or cohorting of MRSA colonised patients with a control group. The study took place in a medical-surgical intensive care unit of a tertiary adult hospital between May 21^st^ 2007 and September 21^st^ 2009. The primary outcome was the rate of MRSA acquisition. A segmented regression analysis was performed to determine the trend in MRSA acquisition rates before and after the intervention.

**Findings:**

The rate of MRSA acquisition was 18.5 per 1000 at risk patient days in the control phase and 7.9 per 1000 at-risk patient days in the intervention phase, with an adjusted hazard ratio 0.39 (95% CI 0.24 to 0.62). Segmented regression analysis showed a decline in MRSA acquisition of 7% per month in the intervention phase, (95%CI 1.9% to 12.8% reduction) which was a significant change in slope compared with the control phase. Secondary analysis found prior exposure to anaerobically active antibiotics and colonization pressure were associated with increased acquisition risk.

**Conclusion:**

Contact precautions with single room isolation or cohorting were associated with a 60% reduction in MRSA acquisition. While this study was a quasi-experimental design, many measures were taken to strengthen the study, such as accounting for differences in colonisation pressure, hand hygiene compliance and individual risk factors across the groups, and confining the study to one centre to reduce variation in transmission. Use of two research nurses may limit its generalisability to units in which this level of support is available.

## Introduction

Contact precautions and single room isolation are often regarded as the *sine qua non* of prevention of transmission of methicillin-resistant *Staphylococcus aureus* (MRSA) [1], however, this is not universally accepted or practised [2]. Recently, some have questioned strategies that target individual healthcare-associated pathogens, as they are time and resource intensive, compared with using generic population-based interventions, such as hand hygiene, antibiotic stewardship, and care bundles [3], [4]. Many studies conclude that contact precautions are essential for MRSA control [5], [6], although there are a number of studies that cast doubt on the necessity [4], [7], [8] and efficacy [9] of contact precautions to control MRSA. For example, results from the paper proposing that a “program of universal surveillance, contact precautions, hand hygiene, and institutional culture change was associated with a decrease in health care–associated transmissions of and infections with MRSA in a large health care system” have been challenged by mathematical modelling suggesting that the control program could have only contributed marginally to the reduction in infections that was reported [10]. Despite guidelines intended to raise standards of reporting and research, the infection control literature remains methodologically poor with inadequate data and inappropriate analyses common [11]. Only one study that we are aware of has examined the effectiveness of isolation precautions alone. This study found that single room isolation with glove and gown use could modestly reduce MRSA transmission, although the estimate has considerable uncertainty and the study did not account for other factors such as hand hygiene compliance [12].

At our institution, MRSA is endemic. Contact precautions are not usually used when managing MRSA colonised patients. Neither active surveillance nor contact precautions are mandated in the state of Victoria. We performed a quasi-experimental research study in our intensive care unit (ICU) population between May 21^st^ 2007 and September 21^st^ 2009. Our hypothesis was that MRSA acquisition would be reduced if single room isolation (or cohorting) accompanied by use of gowns and gloves for MRSA colonized/infected patients (identified via active surveillance using rapid detection methods) were used in an ICU compared with a pre-intervention control group in whom these precautions were not used.

## Methods

### Design overview

This study was a planned, prospective interrupted time series, with a pre-specified date for change in management after 14 months of study, not related to outcome measures. The study was formally implemented as a research study with predefined protocol and endpoints and was initiated by the researchers with no institutional imperative. The study was performed to answer one specific question and the intervention was not influenced or triggered by rates of MRSA, clinical outcomes or any other unspecified influence. The study took place between May 21^st^ 2007 and September 21^st^ 2009, with a change-over date of 21^st^ July 2008.

### Setting and participants

The Royal Melbourne Hospital (RMH) is a university-affiliated tertiary adult hospital with 350 beds. The medical-surgical ICU consists of 24 beds, six single and 18 open-bay. Surgical patients, consisting of trauma, cardiothoracic, neurosurgery, and general specialities, comprise 70% of ICU admissions. Other specialities at RMH include haematology/oncology, bone marrow transplantation and renal transplantation. Post-operative cardiothoracic surgery patients are admitted to this ICU routinely for monitoring. Nurse-to-patient ratios in ICU are 1∶1 except for patients in step-down care for whom the ratios are 1∶2. The infection control service made regular visits to the ICU during the study period.

Consecutive patients admitted to the RMH ICU during the study period were included. The study observed 4317 patients, admitted 4781 times. The source of admission was from home in 80% of patients, a chronic care institution in 1% and other hospitals for the remainder. The crude mortality rate of patients was 7.6%.

The study was approved by the Melbourne Health Human Research Ethics Committee. The committee did not require that individual patient consent be sought as the study was considered to be a quality improvement initiative.

### Screening and intervention

In both study phases, patients were screened for MRSA, but results were not specifically acted upon in the control phase. Results were available to clinicians but no specific infection control precautions were used; instead patients with MRSA colonization were managed according to usual hospital practice, as follows. Staff were required to wear plastic aprons for all patient contact. Contact precautions were not routinely used when caring for MRSA colonized or infected patients and isolation/cohorting was not practiced. On the rare occasion where a patient was deemed to be likely to shed high quantities of MRSA, such as an MRSA positive wound with discharge that could not be contained, he/she would be cared for with contact precautions and isolation/cohorting, although this almost never occurred. Use of alcohol-based hand rub solution (ABHRS) was hospital policy for hand disinfection. Hand hygiene was actively promoted, with regular compliance monitoring and feedback. MRSA decolonization was not routinely used. Antibiotic management of patients in the ICU was not altered for the purposes of the study.

In the intervention phase, results of MRSA screening were conveyed to the ward by telephone and colonized patients were cared for using contact precautions and isolation/cohorting, detailed in Table 1. The only change during the study period was introduction of the study intervention. During both phases of the study, research nurses were employed to ensure compliance with swabbing, contact precautions and isolation/cohorting as well as data collection. During most of the study, this involved seven days per week of nursing time.

**Table 1:** Study characteristics and intervention.

**Setting:** 24 bed medical-surgical ICU in adult tertiary hospital		**Dates:** 21^st^ May 2007 to 21^st^ September 2009	**Population characteristics:** Unselected consecutive ICU admissions. Endemic MRSA.	
**Major intervention:** Change from usual care of MRSA colonized/infected patients (standard precautions and single-use plastic aprons as for all ICU patients) to active surveillance, single room/cohorting plus long-sleeved gowns and gloves (contact precautions) for MRSA colonized/infected patients				
	**Active surveillance**	**Results**	**Contact precautions**	**Isolation policy**
**Phase 1 (Control Phase)** 21/5/2007–20/7/2008	Screening for MRSA (nose, throat, groin, axilla)		Aprons for all patients, no contact precautions for MRSA patients*	No isolation for MRSA patients unless shedders *
	Culture only			
	Results available on electronic pathology records but not actively communicated or specifically acted upon			
**Phase 2 (Intervention Phase)** 21/7/2008–21/9/2009	Screening for MRSA (nose, throat, groin, axilla)		Contact precautions (long-sleeved gowns, gloves) for MRSA patients	Single room or cohorting with other MRSA patients
	Culture+PCR			
	Results rung through to nurse in charge as soon as available			
**Antibiotic policy (both phases):** No change during study period				
**MRSA decolonization policy (both phases):** Not used during study period				
**Pre-emptive isolation (both phases):** Not used during study period				
**Hand hygiene policy and intervention:** Alcohol based hand rub in all patient cubicles. Regular hand hygiene audits that did not change during the study period.				
**Primary study outcome:** Proportion of patients that acquired MRSA, MRSA attack rate (new acquisitions per 1000 at-risk patient days)				

* Unless on risk assessment deemed to be a high-shedder, where contact precautions would be used (but rarely enacted).

Nose, throat, groin, and axilla swabs were taken on admission, Monday, Thursday and discharge (or within 48 hours of discharge) in both study phases. Swabs were cultured in both phases. In the intervention phase, swabs were additionally processed using polymerase chain reaction (PCR). Culture results were used to determine the study outcome (MRSA acquisition) in both phases. PCR results were used only to determine isolation status of patients during the intervention phase and were not used in outcome calculations to ensure that detection bias did not occur if PCR and culture had different sensitivities for detecting MRSA. If a patient was detected to be MRSA positive either via clinical culture or screening swab culture, this was also used to determine isolation status (although most patients that were positive on clinical culture had already been detected using PCR and were thus already in contact precautions). During the intervention phase, positive results obtained via PCR or via culture were telephoned through to the ICU when available and patients were placed in contact precautions as soon as possible.

### Hand hygiene and infection control compliance monitoring

Observations of compliance with hand hygiene and infection control precautions were made for one hour per week on most weeks throughout the study. Hand hygiene compliance monitoring, based on methodology used in other studies [8], was the proportion of perceived opportunities in which appropriate hand hygiene was observed. For infection control observations, compliance was reported as the proportion of times healthcare workers used gloves or long sleeved gowns on each occasion that the precautions were indicated, appropriate to the task or additional precautions that were being used for each patient.

### Microbiologic methods

In both study phases, patients were swabbed using BD culture swabs™ in liquid Stuart's medium (Becton Dickinson). Cultures were performed on chromogenic MRSA media (Chrom-ID MRSA™ bioMérieux). Identification of *S. aureus* was based on production of heat stable DNase, supplemented by a secondary mannitol fermentation agar plate. Sensitivity testing was performed using agar dilution.

In the intervention phase, swabs were cultured using the same methods as in the control phase. Nose and groin swabs were also processed using the IDI-MRSA assay (Infectio Diagnostic, Inc., Sainte-Foy, Quebec, Canada) with the Smart Cycler II rapid DNA amplification system (Cepheid, Sunnyvale, CA) [13].

In order to determine the relatedness of isolates and whether isolates were clustered, they were typed using pulsed field gel electrophoresis (PFGE). MRSA screening isolates from both study phases were frozen at −80°C. PFGE of *Sma*1 digested genomic DNA of the isolates was performed as described previously [14]. Digital images of the gels were analysed with GelCompar software (Applied Maths, Belgium). Isolates with similarity of 80% or more were considered a clonal cluster.

### Definitions

MRSA acquisition was defined in three ways:

1. New colonization using screening swabs only: conversion from negative to positive in patients who had ≥2 sets of screening swabs taken.
2. New colonization using screening and clinical specimens: initial negative screening swabs and subsequent positive screening or clinical specimens in patients who had one set of screening swabs and a subsequent clinical sample or screening sample.
3. New infection: one negative screening swab and subsequent infection in patients who had at least one negative set of screening swabs.

A clinical specimen was defined as one sent on clinical indication. If a patient had known MRSA found on a clinical specimen in the six months prior to ICU admission, they were not included as an acquisition even if their first set of swabs was negative.

At risk patient day was defined as a day in which one patient who was not MRSA colonised or infected spent on the ward, for whom screening swabs were performed before and after.

MRSA infection was determined using Australian Infection Control Association definitions; that is, a sterile site isolate or a non-sterile site clinical isolate and MRSA specific antibiotic therapy administered by a clinician [15]. Infections were determined by the research nurse with review by one of the investigators (CM). Daily colonization pressure was defined as high if two or more patients in the ICU had MRSA colonisation or infection.

### Sample size estimation

Based on a known 6.9% MRSA colonization prevalence at RMH ICU discharge [17], of which 60% are estimated to have been acquired during the ICU stay (extrapolating from a previous study at another Melbourne hospital) [18], we calculated 1239 patients were needed in each group to detect a decrease in acquisition from 4% to 2% with 80% power. We planned to screen 1500 patients in each group, inflating the study numbers to allow for loss of precision in the estimates owing to fluctuations in prevalence (due to serial dependence) [19] during the study. The study had an 80% power to detect a significant difference in hazard with a two tailed P value of 0.05, assuming a hazard ratio of 0.5. In order to achieve this power, we required a minimum of 50 events in the first phase. As such we determined to continue the pre-intervention observation phase until at least 12 months or at least 50 acquisitions were observed, whichever was the longer period. A review at 11 months determined the threshold of 50 acquisitions had not been reached and both phases were reset to a 14 month duration. The final number of individuals screened at least twice, and hence included in the analysis, exceeded 1800 in each group.

### Statistical analysis including outcomes

Primary analysis was the hazard ratio; pre- and post- intervention for the individual hazard of MRSA, defined as acquisition per *at-risk patient day*, after adjusting for pre-defined covariates. Time-varying covariates (exposure to antibiotics, time since admission, colonization pressure, hand hygiene compliance, bed occupancy, phase of study, patient-to- nurse ratio) and time-constant covariates (age, gender, APACHEII score, medical unit) were chosen based on findings from previous studies [8], [9], [16], [18], [20], [21], [22], [23] and specified *a priori*.

Each individual in the study was analysed as an independent study participant. The assumed independence was conditional on the covariates for each participant, including daily colonization pressure. Incorporating time-varying covariates required daily updating of these covariates for each patient. The hazard of MRSA acquisition was calculated for each patient day. The hazard on day *t* for patient ‘*i*’ is given by
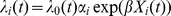
where *X_i_ (t)* is the vector of covariates for individual *i* on day *t* and *β* is the vector of coefficients. λ_0_ (t) is the baseline hazard on day *t* (the hazard if all other variables are zero) and α_i_ is the shared frailty of individual *i* over all of that individual's admissions. A proportional hazards assumption was used in the analysis and test of this assumption made using Schoenfeld residuals [24], showing proportional hazards could be assumed (P = 0.30). The primary outcome for this study was the hazard ratio for the effect of the intervention phase on an at-risk individual's daily risk of MRSA acquisition, after adjusting for covariates. Analysis was performed using STATA/SE 11.0 (StataCorp LP).

### Segmented regression analysis

We used a model of segmented regression, allowing for a step change and the time of the intervention, and estimated parameters based on an assumed proportional reduction (or increase) over time. Our modelled incidence per 1000 at-risk patient days on month *m* is given by *inc(m) = inc (m−1) p_c_* for the control phase, where p_c_ is the proportional change each month in the control phase and *inc (m) = inc (m−1) p_i_* for the control phase, where p_i_ is the proportional change each month in the intervention phase. At two points in the time series, the incidence was assumed not to depend on the previous month's incidence, namely *inc*(1) = *a* the intercept, the incidence at the start of the study, and is estimated as a separate parameter, and *inc (15) = b* is the estimated starting incidence of the intervention phase.

The parameter estimates were made using a Bayesian inference framework, which allowed for estimation of functions of parameters, including estimated incidence at the time of the intervention (*a p_c_^14^*) and the end of the intervention (*b p_i_^14^*) and the estimated difference between them. Non-negative uniform priors were assumed for all parameters and a Poisson error function for the observed incidence (given the modelled incidence) was used to construct the likelihood function. Burn-in of 10000 iterations and parameter acquisition of 90000 iterations was used.

### Analysis of Staphylococcal bacteraemia

Using the same methodology as described above, we analysed the rates of MRSA and MSSA bacteraemia in the hospital. We assessed for changes in incidence both at the time of the intervention and over time following the intervention.

## Results

Demographics, testing frequency, and patient characteristics other than the intervention and outcome were very similar in the two groups (Table 2). Patients had an average age of 57.6 years and an average length of ICU stay of 3.4 days. A slightly higher admission prevalence of MRSA-positive patients was found in the intervention phase group (5% versus 4.5%). The average time from taking specimen to culture notification was 73.6 hours (median 68.5, range 26.42–157.3) (n = 91) and from taking specimen to PCR notification was 19.7 hours (median 18.4, range 3.08–52.92) (n = 213).

**Table 2:** Patient characteristics.

	Phase 1	Phase 2
	2183 patients	2196 patients
	2387 admissions	2394 admissions
**Average age (years)**	(n = 2377)	(n = 2381)
**(median, range)**	57·6	57·7
	(61, 15–98)	(61, 15–101)
**Average ICU length of stay (days)**	(n = 2387)	(n = 2394)
**(median, range)**	3·2	3·4
	(2, <1–75)	(2, <1–86)
**Gender**	(n = 2387)	(n = 2392)
Female	868 (36·4%)	841 (35·2%)
**Medical Unit**	(n = 2374)	(n = 2359)
CT surgery	717 (30·2%)	688 (29·1%)
Medical	784 (33·0%)	768 (32·6%)
Other surgery	525 (22·1%)	536 (22·7%)
Trauma	348 (14·7%)	367 (15·6%)
**Median APACHEII**	(n = 2370)	(n = 2374)
**(range)**	13 (0 to 47)	14 (1 to 50)
**Percentage of time MRSA colonized patients in contact precautions**	7·3%*	76·4%
**Percentage of time MRSA colonized patients in single rooms or cohorted**	18·5%*	46·5%

* Patients were in contact precautions or single room/cohorted for reasons other than MRSA.

Denominators may vary if data were missing.

Swab results are shown in Table 3. Almost all patients had some screening swabs taken and 77% had at least 2 sets taken, hence were able to be included in the study. In the control phase, 2.7% of patients acquired MRSA, compared with 1·3% in the intervention phase.

**Table 3:** Swab results.

**≥2 sets of swabs taken per admission**	(n = 2374)	(n = 2366)
	1819 (76·6%)	1822 (77·0%)
**Mean number of swabs taken (median, range)**	(n = 2353)	(n = 2346)
	2·3 (2, 1–24)	2·4 (2, 1–27)
**Screening swabs taken**	(n = 2387)	(n = 2394)
	2374 (99·5%)	2366 (98·8%)
**MRSA positive at admission (previous +ve or screening swab +ve)**	(n = 2374)	(n = 2366)
	108 (4·5%)	118 (5·0%)
**Any MRSA screening/clinical sample positive**	(n = 2374)	(n = 2366)
	155 (6·5%)	126 (5·3%)
**Infections caused by MRSA**	(n = 2353)	(n = 2343)
	29 (1.2%)	30 (1.3%)
**Number of MRSA acquisitions**	(n* = 2173)	(n* = 2135)
	58 (2·7%)	27 (1·3%)
**Rate of acquisition (per 1000 at risk patient days)**	(n** = 3136)	(n** = 3430)
	18·5	7·9

* Patients at risk.

** Days at risk.

Denominators may vary if data were missing.

### Culture and PCR results

There were 5589 swab sets that were processed by culture and PCR. Of these, 5000 (89.46%) were both culture and PCR negative, with 158 (2.83%) both positive, 42 (0.75%) culture positive and PCR negative, 159 (2.84%) culture negative and PCR positive, 2 (0.04%) culture positive and PCR indeterminate, and 228 (4·08%) culture negative and PCR indeterminate.

71.6% of 239 isolates tested were resistant to erythromycin, with 26.4% sensitive and 2% intermediate. Of the isolates tested, 79.5% of 239 were resistant to ciprofloxacin, with 20.2% sensitive and the remainder intermediate.

### PFGE results

**Figure 1:**
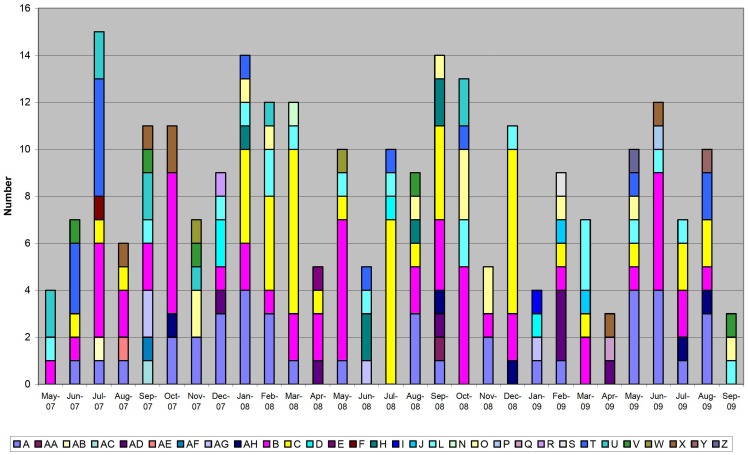
PFGE subtypes.

PFGE was performed on 255 isolates from 230 patients (Figure 1). There were 19 patients with more than one MRSA isolate typed, with five having more than one PFGE type and none more than two different types. Overall there were 31 PFGE types and Figure 1 shows different types present each month. The most frequently encountered types were A [39/255 (15.3%)], B [55/255 (21.6%)], C [46/255 (18.0%)], L [20/255 (7.8%)], T [15/255 (5.9%)], O [14/255 (5.5%)], and U [10/255 (3.9%)]. We intended to review PFGE type in cases in which a putative transmission event occurred (two cases of MRSA in which at least one was a new acquisition with overlapping time in ICU). Unfortunately there were insufficient numbers of these events for any inference to be valid.

### Hand hygiene and infection control compliance

There were 6179 hand hygiene compliance opportunities observed in 351 sessions. Compliance ranged between 12 and 34%. There was a very slight, non-significant trend upwards throughout the study (see Figure S1). Compliance with infection control precautions was observed in 3761 opportunities in 342 sessions. Gloves were worn when required in 698/795 (87.8%) observations in the control phase and in 622/722 (86.2%) in the intervention phase (P = 0.3). Long sleeved gowns were worn when required in 109/120 (90.8%) opportunities in the control phase and 208/235 (88.5%) in the intervention phase (P = 0.5).

### Primary analysis

After accounting for the *a priori* determined covariates given in Table 4, the hazard ratio of MRSA acquisition in the intervention phase was significantly lower than in the control phase, with a hazard ratio of 0·39 during the intervention compared with control (95% CI 0.24 to 0.62).

**Table 4:** Hazard ratios for covariates associated with MRSA acquisition.

	Univariate Analysis				Multi-variate analysis			
Predictor Variable	Hazard Ratio	P-value	95%CI		Hazard Ratio	P-value	95%CI	
**Intervention phase**	0.42	<0.001	0.26	0.66	0.39	<0.001	0.24	0.62
**Age >70**	1.03	0.92	0.63	1.67	1.13	0.63	0.68	1.87
**Gender = male**	1.36	0.20	0.85	2.16	1.44	0.12	0.91	2.29
**Apache score >18**	0.96	0.86	0.61	1.51	0.93	0.77	0.59	1.48
**Cardiothoracic patient**	0.41	0.04	0.17	0.97	0.54	0.23	0.20	1.47
**Patient to Nurse Ratio higher than 1∶1**	1.03	0.93	0.51	2.07	1.20	0.63	0.57	2.53
**Number of beds occupied>25**	0.59	0.05	0.35	0.99	0.77	0.34	0.45	1.31
**Colonization pressure high: 2 or more**	1.77	0.01	1.14	2.73	1.73	0.02	1.11	2.69
**Prior exposure to:**								
**Penicillin/Amoxycillin**	1.10	0.77	0.58	2.08	0.79	0.45	0.43	1.45
**Anaerobic activity**	2.44	<0.001	1.60	3.73	2.42	<0.001	1.52	3.86
**Broad Spectrum**	0.84	0.51	0.51	1.40	0.85	0.54	0.51	1.42
**MSSA active agent**	0.61	0.16	0.30	1.22	0.78	0.53	0.36	1.68
**MRSA active agent**	0.84	0.46	0.54	1.33	1.05	0.85	0.66	1.66
**Quinolones**	0.87	0.79	0.32	2.39	0.77	0.62	0.27	2.17
**Cephalosporins**	1.31	0.33	0.76	2.24	1.08	0.79	0.59	1.98

### Secondary analysis

On univariate analysis, being in the control phase of the study, prior exposure to anaerobically active antibiotics and high ward colonization pressure were associated with increased daily risk of MRSA acquisition. Higher numbers of beds occupied on the ward and being a cardiothoracic surgical patient was associated with reduced MRSA acquisition. On multivariate analysis, being in the control phase of the study, prior exposure to anaerobically active antibiotics and high ward colonization pressure continued to be significantly associated with increased daily risk of acquisition. All other covariates were non-significant, as shown in Table 4.

### Segmented regression

**Figure 2:**
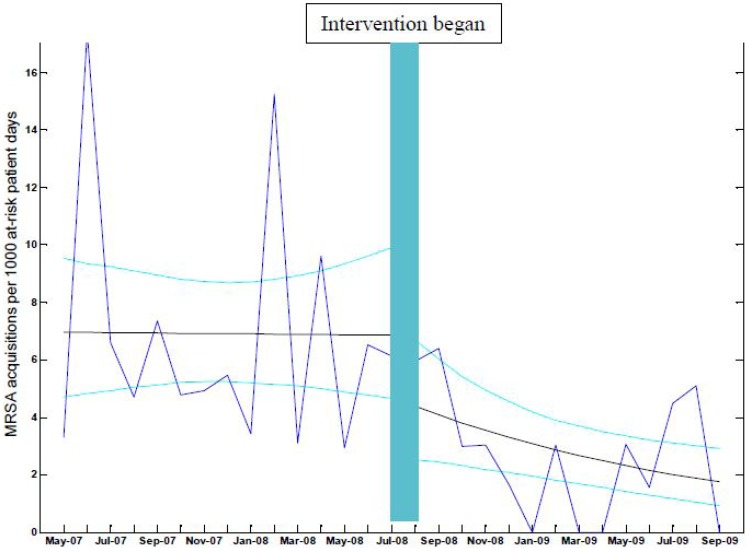
Segmented regression model. Incidence of MRSA per 1000 at risk patient days in ICU over the study period. There was a decline in MRSA acquisition of 7% per month in the intervention phase, which was a significant change in slope compared with the control phase (95% CI for change in slope 1.9 to 12.8% reduction). The dark blue lines are the data, the black lines are the model best fit results and the light blue lines are the upper and lower values of the 95% credible intervals for the model estimates.

Figure 2 shows the monthly incidence of MRSA incidence over the study. The mean number of MRSA acquisitions per month was 3.0, variance 5.4. The value of the variance on the mean of 1.8 supports extra-Poisson variation. We used a model of segmented regression, allowing for a step change at the time of the intervention, and estimated parameters based on an assumed proportional reduction (or increase) month by month. The incidence of MRSA acquisition is estimated to have been approximately flat during the control phase of the study, at 7 acquisitions per 1000 at-risk patient days (estimate of monthly decrease of 0.1%, 95% CI = 3.5% reduction to 4.5% increase each month). There was an observed decline in incidence during the intervention phase of 7% each month, which was statistically significant (95% CI = 12.8% reduction to 1.9% reduction). At the time immediately after the introduction of the intervention, we estimate that the number of acquisitions reduced by 2.1 per 1000 at-risk patient days which was not statistically significant (95% CI −6.0 to +1.6 per 1000 at risk patient days).

### Comparison of nosocomial MRSA and MSSA bacteraemia rates during the study period

During the study period, data on *Staphylococcus aureus* bacteraemias were collected in the ICU and throughout the hospital. Numbers were too small for meaningful comparison in the ICU however the hospital-wide rates were examined and are given in the Figures S3 and S4. The rates of nosocomial MSSA bacteraemia throughout the hospital were 7.0 per 10,000 patient days, with a trend toward an increase in MSSA bacteraemia in the intervention phase of the study (no change in slope null hypothesis P = 0.06). The rates of nosocomial MRSA bacteraemia were stable at approximately 2.3 per 10,000 patient days (no change in slope null hypothesis, P = 0.6).

### Additional analyses

The File S1 contains the sensitivity analysis. Figure S1 reports hand hygiene throughout the study period and Figures S2 and S3 report fidelity of the hospital staff to study interventions. Figures S4 and S5 show rates of MRSA and MSSA bacteraemia.

## Discussion

We found a 60% reduction in acquisition of MRSA in the ICU during an intervention in which MRSA colonized patients were identified using rapid molecular detection methods and managed using contact precautions and isolation/cohorting. This study is unique as it tested a single intervention that was instituted as a planned prospective research study, with the analysis taking into account serial dependence that inevitably occurs when studying communicable diseases in a confined setting. Segmented regression demonstrated that there was no trend in MRSA acquisition rates prior to the intervention phase, but there was both a drop in acquisition rates immediately after the intervention and further decline during the intervention phase.

The secondary findings from this study are of interest, but are not intended to suggest causal associations. Anaerobically active agents were the only antibiotic class that was associated with MRSA acquisition in the multivariate analysis. This is unexpected, but may suggest an effect on resident flora, as has been shown with VRE [25]. The association of high bed occupancy with reduced MRSA acquisition warrants further consideration. High ward occupancy may lead to fewer contacts per patient which has been predicted by some models to lead to lower transmission of infection [23].

PFGE of the MRSA isolates showed the presence of multiple clones with several predominating at different times. Therefore there appears to be no outbreak of a single PFGE type during the study, but rather importation (and possibly onward transmission) of several types. This is not unexpected given the relatively low acquisition rate compared with the admission prevalence.

We employed research nurses to optimise compliance with the intervention. This however, means that the results may not be generalizable to other institutions where this level of support is not available.

A number of studies have examined the utility of active surveillance with conflicting results [26], [27], but we have found no studies testing contact precautions in the context of rapid detection as a single intervention in a prospective, systematic way. Most of the publications reporting effectiveness of contact precautions used them as one of multiple serial interventions in observational outbreak reports [28], [29]. One previous study found that patients in single room isolation had no less MRSA acquisition than those cohorted, but that study did not incorporate rapid diagnosis and contact precautions [21].

A recent cluster randomised trial found that use of active surveillance with expanded contact precautions did not reduce transmission of MRSA [9]. Although this methodology is regarded as the optimal study design, that study had high baseline variation in transmission rates and delays in screening, which reduced its power. Although our study was only performed at a single centre, it took into account serial dependence, bias and clustering in the analysis by incorporating colonization pressure into the daily hazard analysis.

It is interesting to speculate how this intervention is associated with reduction in MRSA acquisition. In the intervention phase, MRSA patients spent 76.4% of their time in contact precautions and 46.5% in single rooms from the date of their first positive swab (or admission date if they were known to be colonized). Although this was less than the study aimed to achieve, the time in contact precautions was substantially higher than in the control phase, and it may have been sufficient to account for a reduction in transmission, akin to models examining improved but imperfect hand hygiene compliance [30]. The study design cannot exclude temporal effects or a Hawthorne effect. However, it should be noted that the number of colonized patients entering the ICU was slightly higher in the intervention phase than the control phase, that hand hygiene compliance did not significantly change and no other interventions took place during the study.

A finding of efficacy of rapid detection and isolation/cohorting and contact precautions does not lead the authors to conclude necessarily that this intervention is essential. The costs, labour, and time requirements of this intervention were considerable. A cost-benefit analysis is currently being performed to evaluate this intervention compared with other general intervention measures. Adverse effects of isolation are not insignificant and need to be taken into account as well.

The value of the current pathogen-specific intervention is likely to be contingent on local context such as background rates of MRSA colonization and infection and community-associated MRSA rates and ability to detect MRSA and institute contact precautions rapidly. The most recent data reported to the National Healthcare Safety Network showed that MRSA was responsible for only 8% of device- and procedure- associated healthcare-associated infections (HAIs) [31]. The intervention in this study was 60% effective, having a potential effect of reducing total HAIs by only 5%. Thus, any intervention directed at MRSA alone will only have a small effect on the total number of HAIs, as has been suggested previously [3].

Generic population measures that reduce the incidence of HAIs caused by all organisms, such as enhanced hand hygiene, antibiotic stewardship, environmental cleaning and bundles of care [32], have greater potential to reduce HAIs. The authors recommend that proven generic measures be adopted as a priority and the decision to adopt pathogen-specific screening and contact isolation be made according to local context including relative burden of MRSA and available resources.

## Supporting Information

Figure S1 **Hand hygiene compliance throughout the study period.**
(TIF)

Figure S2 **Use of single rooms or cohort over the study period.** The Y axis shows the number of colonized patients in the ward as the height of the stacked bar plot. The colonized patients put into single rooms or cohorted are shown in blue, while those not in single rooms are shown in red.
(TIF)

Figure S3 **Use of contact precautions over the study period.** The Y axis shows the proportion of patients in contact precautions over the study period.
(TIF)

Figure S4 **Rate of MSSA infection in the hospital during the study period.** There was a non-significant trend to increasing MSSA over the study period P = 0.06.
(TIF)

Figure S5 **Rate of MRSA infection in the hospital during the study period.** The rates of MRSA bacteraemia were stable P = 0.6.
(TIF)

File S1 **Supporting information file. The Supporting Information file contains the sensitivity analysis.**
(DOCX)
