# Supplementary figures and images for: Do Active Surveillance and Contact Precautions Reduce MRSA Acquisition? A Prospective Interrupted Time Series
Source: PLoS One. 2013 Mar 21;8(3):e58112. doi: 10.1371/journal.pone.0058112 (PMC3605415; doi:10.1371/journal.pone.0058112)

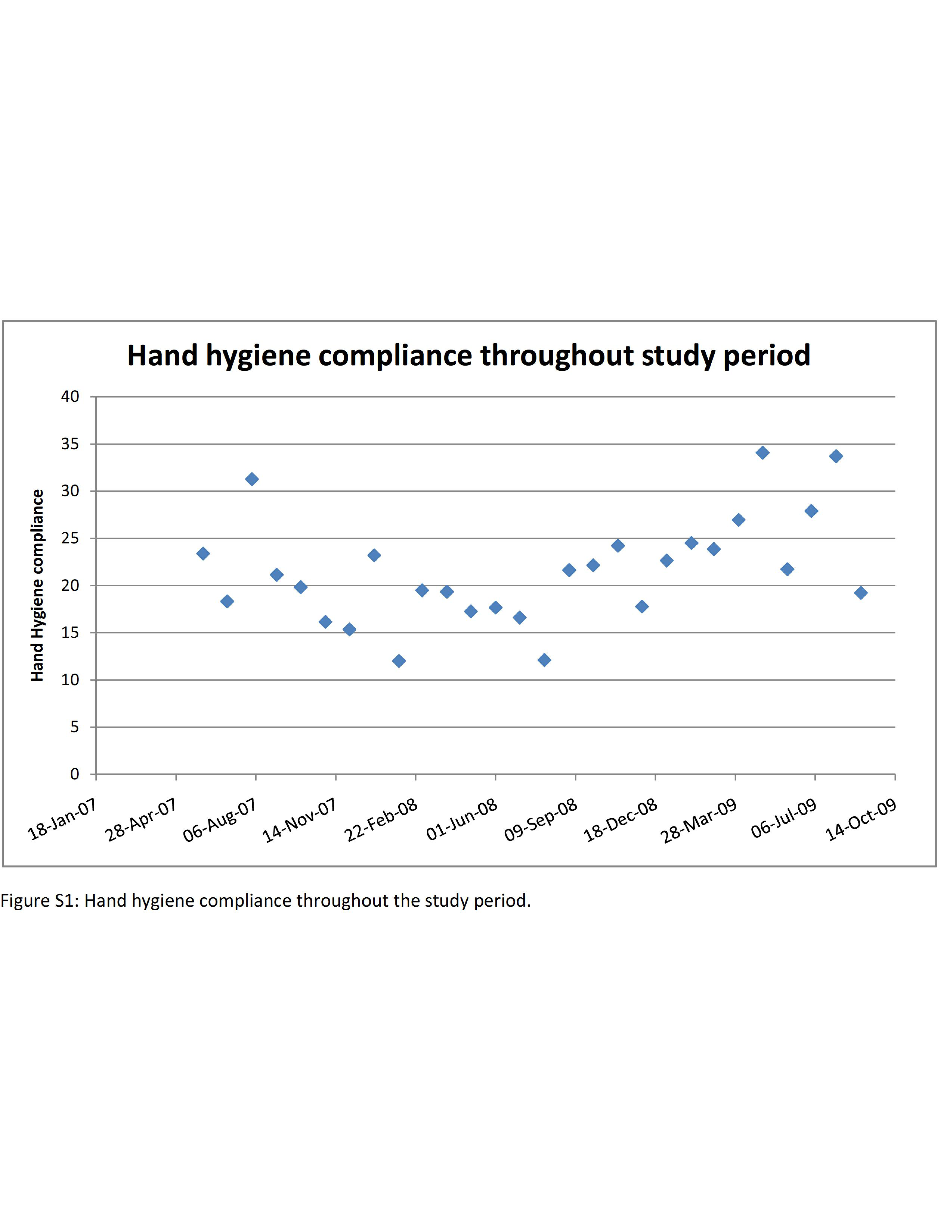

Supplement: Figure S1 — Hand hygiene compliance throughout the study period. (TIF) [file pone.0058112.s001.tif]

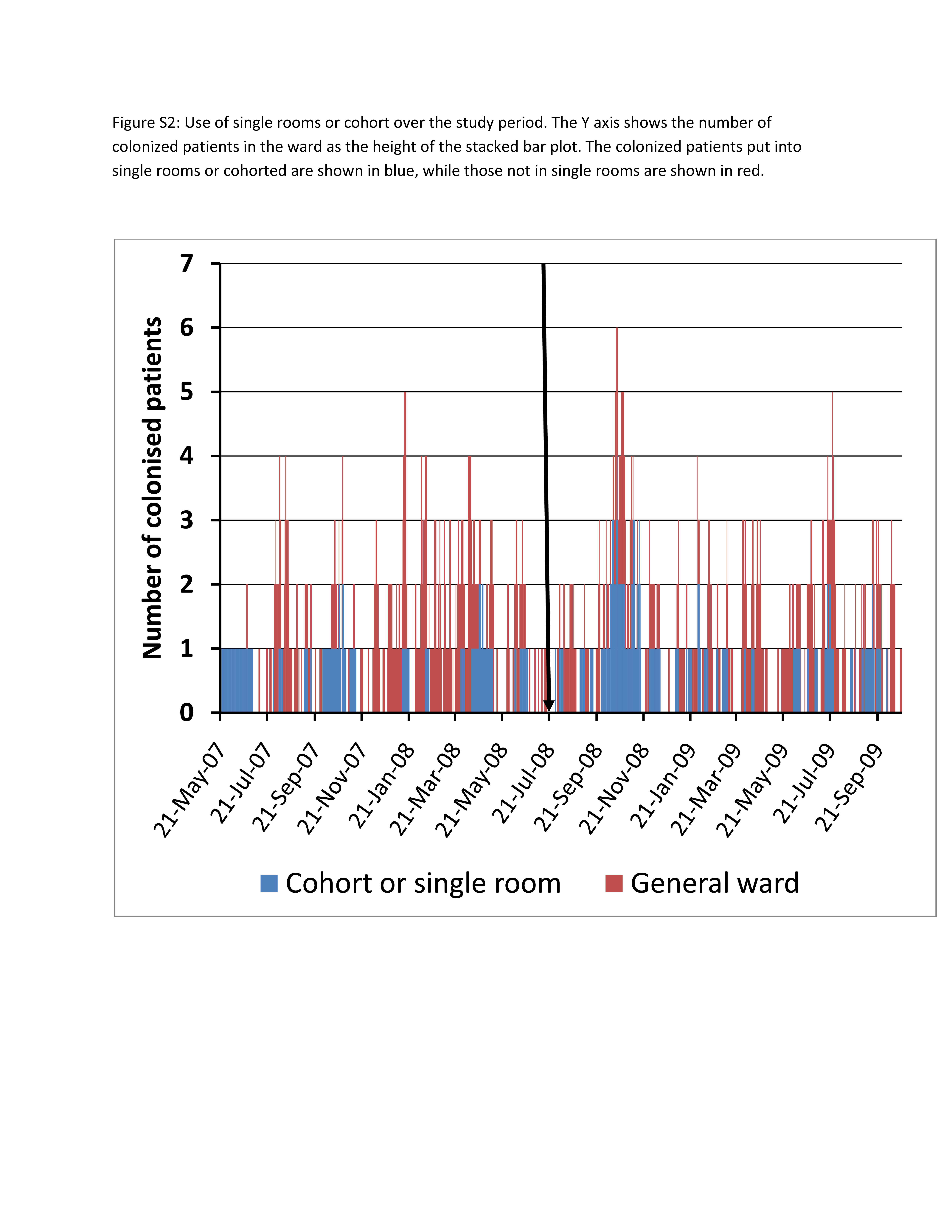

Supplement: Figure S2 — Use of single rooms or cohort over the study period. The Y axis shows the number of colonized patients in the ward as the height of the stacked bar plot. The colonized patients put into single rooms or cohorted are shown in blue, while those not in single rooms are shown in red. (TIF) [file pone.0058112.s002.tif]

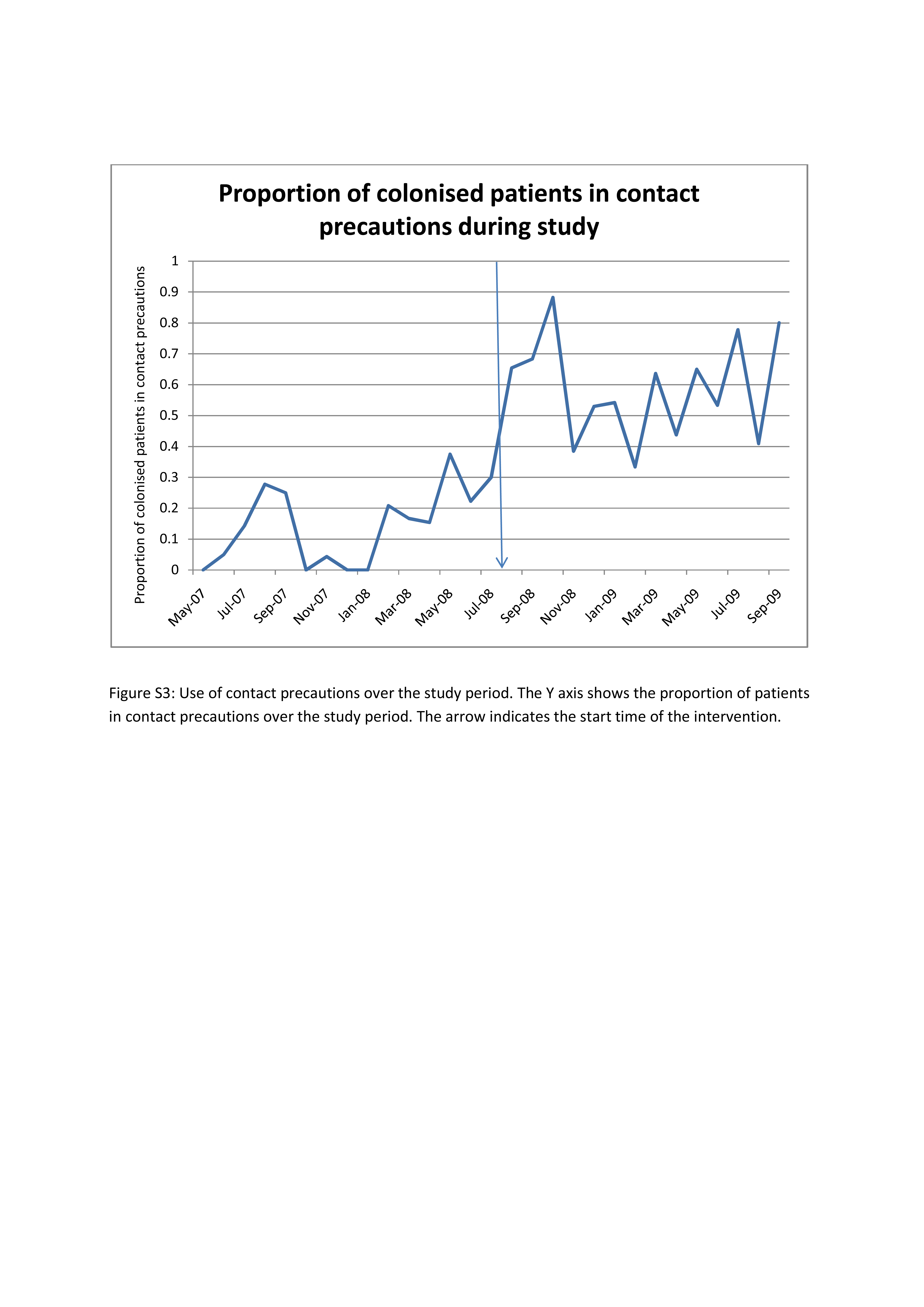

Supplement: Figure S3 — Use of contact precautions over the study period. The Y axis shows the proportion of patients in contact precautions over the study period. (TIF) [file pone.0058112.s003.tif]

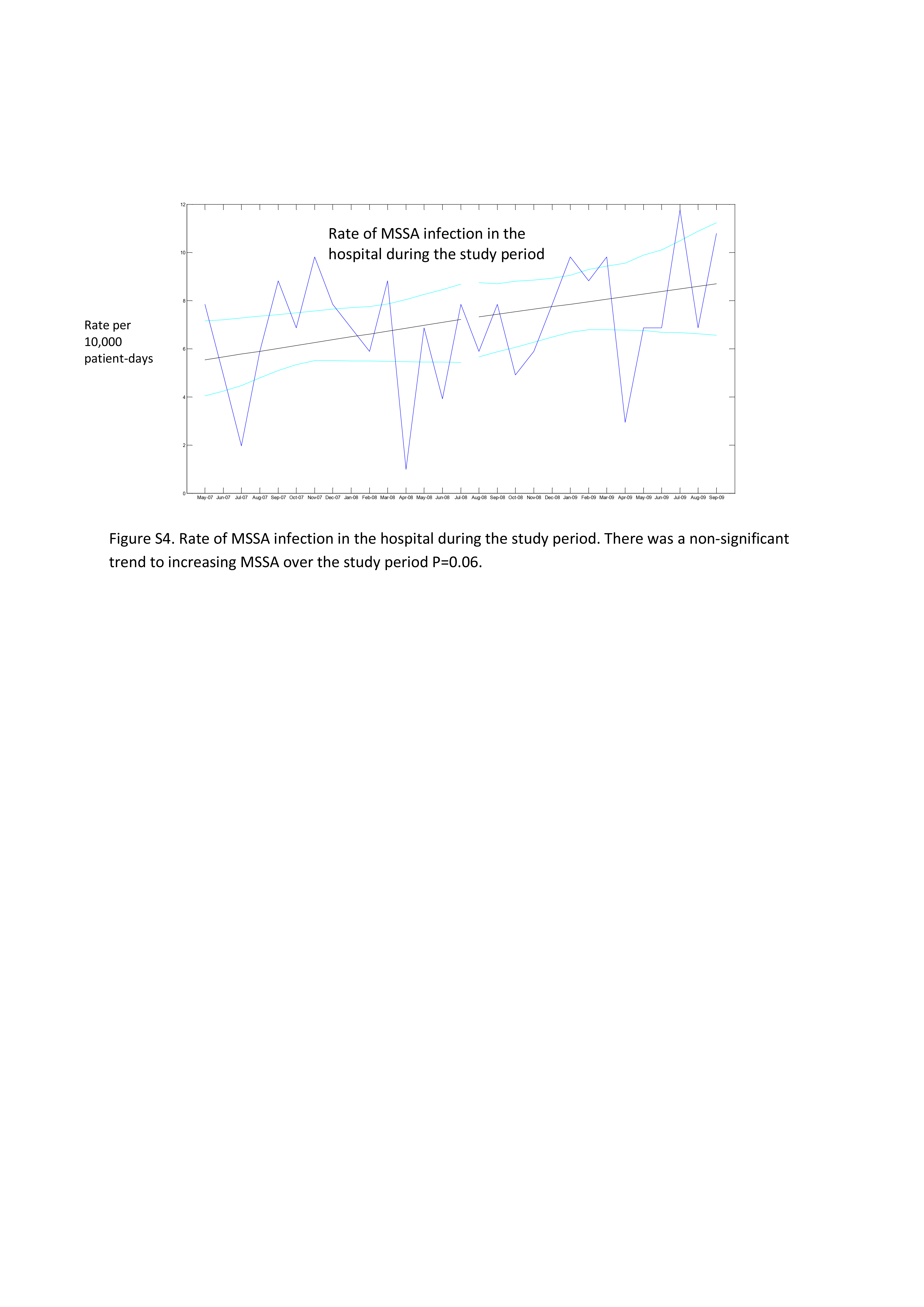

Supplement: Figure S4 — Rate of MSSA infection in the hospital during the study period. There was a non-significant trend to increasing MSSA over the study period P = 0.06. (TIF) [file pone.0058112.s004.tif]

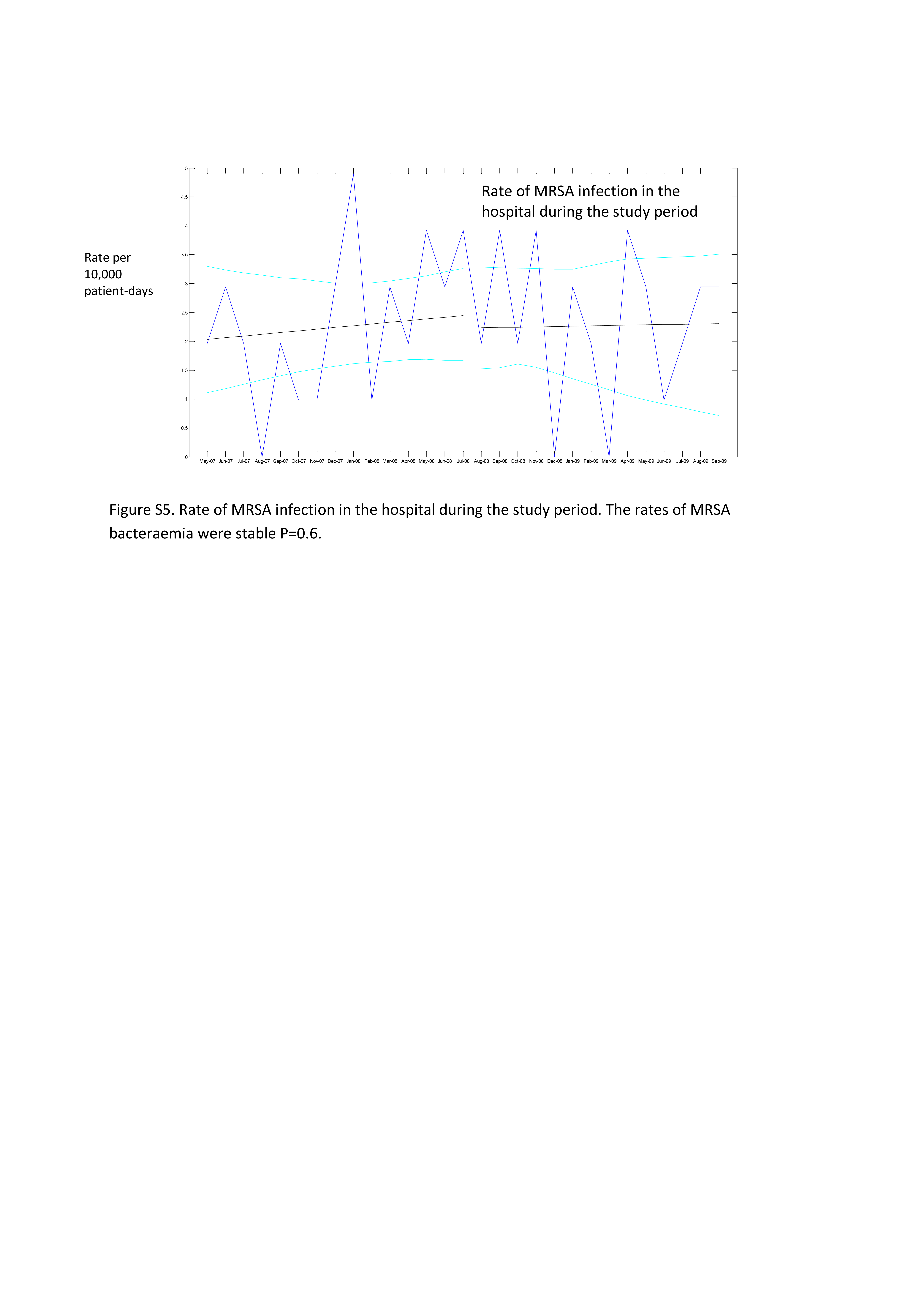

Supplement: Figure S5 — Rate of MRSA infection in the hospital during the study period. The rates of MRSA bacteraemia were stable P = 0.6. (TIF) [file pone.0058112.s005.tif]
