# Supplementary material for: Do Active Surveillance and Contact Precautions Reduce MRSA Acquisition? A Prospective Interrupted Time Series
Source: PLoS One. 2013 Mar 21;8(3):e58112. doi: 10.1371/journal.pone.0058112 (PMC3605415; doi:10.1371/journal.pone.0058112)
Supplement: File S1 — Supporting information file. The Supporting Information file contains the sensitivity analysis. (DOCX) [file pone.0058112.s006.docx]

***Supporting information***

**Sensitivity analysis**

We examined the dataset to ensure the finding of an effect of the intervention phase was robust to the assumptions of the *a priori* model. That is, that the effect of pre- versus post- intervention was not altered by, for example, the model of colonization pressure and its interaction with the phase of the study. We therefore examined the data without colonization pressure included and with colonization pressure expressed as the log of the number of patients colonized; which equates with the Reed-Frost (or mass-action) assumption of colonization pressure. We also examined the effect of colonization pressure exerted by colonised patients in contact precautions and not in contact precautions.

Keeping all other covariates in the model and removing colonization pressure from the model led to little change in effect of phase on acquisition. The hazard ratio for being in the intervention phase was 0.39 (95%CI 0.25 to 0.63). Adding to this model the number of colonized patients to which each individual is exposed expressed as a log value, gives the following results effect of intervention phase 0.39 (95% CI 0.23 to 0.64) effect of log(number of patients colonized) is 1.4 (95% CI 1.05 to 1.87). This has an interpretation of “for every time the number of patients colonized is increased by 170% (multiplied by 2.7), this leads to an increase in risk per day of acquisition of 40%.”

Specifying whether colonized patients were in or out of contact precautions leads to the following results: effect of being in intervention phase 0.51 (95% CI 0.27 to 0.95), log (number of colonized patients in contact precautions) 0.32 (95%CI 0.15 to 0.70), log (number of colonized patients not in contact precautions) 1.56 (95% CI 1.06 to 2.28).

We also examined the effect of the intervention when continuous variables; colonization pressure, APACHEII score, age, and occupancy of the ward, were all input into the model as log values; which is equivalent to assuming a linear relationship between these values and the hazard of acquisition per day. The outcome when these were used is an effect of phase of 0.39 (95%CI 0.24 to 0.64).

**Hand Hygiene: fidelity to interventions and to hand hygiene throughout the study period**

Figure S1 demonstrates the proportion of hand hygiene events that took place per hand hygiene opportunity. Improving hand hygiene was not part of this study, and hand hygiene was encouraged throughout the study. Hand hygiene compliance was low throughout the study but comparable with other studies [29]. Compliance rates in this study may have been particularly low because of the stringent criteria used to determine compliance at that time at RMH [6]. On univariate regression, hand hygiene compliance had no effect on acquisition. The lack of effect may have been because measurements of hand hygiene compliance were not representative of the true compliance (this method of measuring compliance, whilst used often, has never been validated) or because there was truly no relationship. It would be interesting to know whether the effect of the intervention would be the same at different levels of hand hygiene compliance.

Figure S2 shows the use of single rooms or cohorting over the study period and Figure S3 shows the use of contact precautions over the study period.

**Rates of MRSA and MSSA bacteraemia**

Hospital-wide rates of MRSA and MSSA bacteraemia were examined during the study period to measure any temporal trends in staphylococcal infection. Figures S4 and S5 show the rates of nosocomial bacteraemia with MSSA and MRSA, respectively, throughout the study period.
